# Pattern of access to cafeteria-style diet determines fat mass and degree of spatial memory impairments in rats

**DOI:** 10.1038/s41598-019-50113-3

**Published:** 2019-09-18

**Authors:** Michael D. Kendig, R. Frederick Westbrook, Margaret J. Morris

**Affiliations:** 1 Department of Pharmacology, School of Medical Sciences, UNSW, Sydney, Australia; 2 School of Psychology, UNSW, Sydney, Australia

**Keywords:** Obesity, Psychology

## Abstract

Repeated ‘cycling’ between healthy and unhealthy eating is increasingly common but the effects of such cycling on cognitive function are unknown. Here we tested the effects of cycling between chow and a cafeteria diet (CAF) rich in saturated fat and refined carbohydrates on fat mass and place recognition memory in rats. Rats fed the chow diet (control group) were compared with groups fed CAF for either: 3 consecutive days per week followed by 4 days of chow, (3CAF:4CHOW group); 5 consecutive days per week followed by 2 days of chow (5CAF:2CHOW group); or 7 days per week (7CAF group). Total days of exposure to CAF were matched between the latter groups by staggering the introduction of CAF diet. After 16–18 days of CAF, spatial recognition memory was significantly worse in the 7CAF group relative to controls. After 23–25 days of CAF, both the 7CAF and 5CAF:2CHOW groups, but not the 3CAF:4CHOW group, were impaired relative to controls, mirroring changes in fat mass measured by EchoMRI. CAF feeding did not affect object recognition memory or total exploration time. These results indicate that even when matching total exposure, the pattern of access to unhealthy diets impairs spatial memory in a graded fashion.

## Introduction

An unhealthy diet is among the leading risk factors for chronic disease and premature death worldwide^1^ as reflected most clearly in the high prevalence of obesity and metabolic disease around the world. Obesity is also correlated with mild cognitive impairments, particularly in tasks assessing aspects of executive function^2,3^, and adequate nutrition is a key protective factor guarding against cognitive decline^4^. Consumption of diets high in saturated fat and refined carbohydrates is associated with poorer cognitive performance on hippocampal-dependent learning and memory tasks in correlational^5^ and experimental studies in people^6,7^. These findings are consistent with a growing literature demonstrating that *ad-libitum* access to high-fat and/or high-sugar diets in rodents impairs performance in tasks assessing hippocampal-dependent forms of cognition, with impairments found after as little as three days of high-fat diet consumption^8^ and five-seven days access to a cafeteria diet containing energy rich foods eaten by people^9^.

Many people do not eat high-fat, high-sugar ‘junk’ foods exclusively; rather they alternate between healthy and unhealthy meals throughout day-to-day life. More defined shifts in diet may occur when people commence diets that exclude certain foods and/or restrict their energy intake. However, abstaining from highly palatable, energy rich foods for long periods is difficult and most diet attempts prove unsuccessful, with dieters returning to unhealthy eating habits. One study estimated that slightly more than one in six overweight or obese individuals were able to achieve long-term weight loss of more than 10% over a 1-year period^10^. Recent data from an Australian population indicate a striking increase in the prevalence of obesity with comorbid disordered eating behaviours such as bingeing and/or excessive restriction of food intake^11^. These results suggest that people who attempt to control energy intake and body weight in modern environments are prone to repeated ‘cycles’ of healthy and unhealthy eating.

Although intermittent consumption of high energy foods characterises the eating habits of many people, relatively little is known about the effects of this pattern of intake on cognition. Results from animal models show that restricting access to high-fat and/or high-sugar foods (i.e., for limited hours per day, or limited days per week) can induce binge-like patterns of consumption^12,13^, persistent responding for valued food rewards despite punishment^14^ coupled with decreased motivation for less-preferred foods^15,16^, and increased immobility in the Forced-Swim Test, a putative index of depression-like behaviour, when the diet is removed^17^. This phenotype is argued to resemble an addiction to palatable food^18–20^ and is associated with changes in brain reward networks^21,22^. Deficits in tasks assessing associative learning^23,24^, spatial memory^25,26^ and goal-directed control over food-seeking behaviour^27^ have also been reported following chronic intermittent access to palatable food.

While access to high energy, palatable food is typically limited to several hours per day in models of bingeing and food addiction, other studies have tested the effects of longer bouts of intermittent cycling, for example, providing such food for 2–3 days per week and chow on the remainder^14–17^. However, studies comparing the effects of intermittent and continuous access to high-energy diets typically commence access simultaneously; hence, cumulative exposure to the diet diverges over time. This means that any differences between continuous and intermittent groups could be due either to the type of access or to differences in total exposure to the diet. To our knowledge, no previous studies have compared the cognitive effects of high energy diets provided continuously or intermittently while matching total duration of exposure.

The present experiment therefore assessed the effects of intermittent versus continuous access to a cafeteria diet on place and object recognition memory in rats while controlling for total exposure to the cafeteria diet. Intermittent access consisted in providing groups of rats with cafeteria diet for three, five, or seven days per week, with only chow being provided on the other days. Critically, at the time of testing total days of exposure to the cafeteria diet was equal across the three groups. We were interested in whether exposure to the cafeteria diet per se impaired place while sparing object recognition memory, relative to a fourth control group fed chow. Additional questions of interest were whether continuous versus intermittent access to the cafeteria diet would exert differential effects on place recognition memory, and whether five days per week access to cafeteria diet would exert greater effects on place recognition memory than three days.

## Methods

### Subjects and housing

Forty-eight experimentally naïve, adult male Sprague-Dawley rats were sourced from Animal Resource Centre (Perth, Australia), weighing 155–175 g on arrival to the laboratory. They were housed three per cage (47 cm length × 29 cm width × 15 cm height) in a temperature- and humidity-controlled room maintained on a 12:12 light:dark cycle (lights on at 0700 h), and handled regularly across a one week acclimation period. Rats were provided with access to chow (Gordon’s, 11 kJ/g, 65% energy as carbohydrate, 22% fat, 13% protein) and water across acclimation and the course of the experiment. All procedures were approved by the UNSW Animal Care and Ethics Committee and were conducted in accordance with the Australian code for the care and use of animals for scientific purposes (8th edition).

### Experimental groups

There were four groups. Two were repeatedly cycled between cafeteria (CAF) and chow diets. One of these received access to CAF for three consecutive days per week and chow for four days, Group 3CAF:4CHOW, a design used to study their effects on feeding patterns, but not cognition, in a previous study from our lab^28^. The other cycled group received five consecutive days per week of CAF and two days of chow, Group 5CAF:2CHOW. The remaining two groups received continuous access to CAF, Group 7CAF, or chow, Chow control.

### Cafeteria diet and food intake schedule

The cafeteria diet (CAF) consisted of chow and a variety of commercially available, sweet and savoury foods high in fat and/or sugar, such as meat pies, dim sims, dog roll, cakes, and biscuits, as well as a 10% sucrose solution (in addition to water). CAF foods were refreshed daily, with a minimum of two sweet and two savoury foods provided each day, and with no one food provided for more than two consecutive days. The 10% sucrose solution was available on every day of CAF access. Care was taken to ensure that access was not limited by providing more food than rats would eat. CAF foods were distributed around the cage bedding; chow pellets were scattered in the same way for group Chow.

Food intake was measured over four 24-h periods each week. For groups 3CAF:4CHOW and 5CAF:2CHOW, food intake was measured on the first and last day of cafeteria diet access, and on the first and last day of chow access. This corresponded to Monday, Wednesday, Thursday and Sunday for group 3CAF:4CHOW, and to Monday, Friday, Saturday and Sunday for group 5CAF:2CHOW. For group 7CAF, consumption was measured on Monday, Wednesday, Friday and Sunday. Food intake in group Chow was measured on Monday and Sunday, with the third and fourth measures taken on Wednesday and Thursday for half the group and on Friday and Saturday for the other half. This was done to match measurements for 3CAF:4CHOW and 5CAF:2CHOW groups, respectively. The three groups exposed to CAF received the same set of CAF foods for the first five food intake measurements.

Food intake measures began at 3–5 pm by weighing individual food and liquid items to the nearest 0.1 g. Twenty-four hours later foods and liquids were weighed again after a careful search of cage bedding for food fragments, and intake was calculated as the difference in weights. Consumption of each food in grams was converted to kJ per manufacturers’ information.

**Figure 1:**
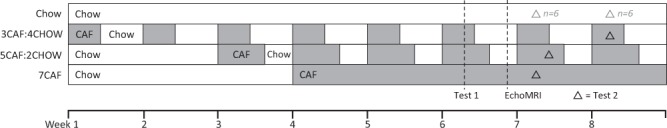
Experimental timeline showing staggered introduction of the cafeteria diet (CAF, shaded). Diet access was staggered such that place and object recognition memory tests were conducted on CAF day 16–18 (Test 1) and Test 2 was conducted at CAF day 23–25 (Test 2; triangle symbol). Rats underwent an EchoMRI scan at the end of week 6 and were euthanised after week 8.

The diet schedule across the experiment is shown in Fig. 1. Importantly, the introduction of CAF diet was staggered so that total exposure to CAF was matched at 16–18 days when the first place and object recognition memory tests were held. Thus, CAF diet cycling commenced in week 1 for group 3CAF:4CHOW, followed by group 5CAF:2CHOW in week 3, and by group 7CAF in week 4.

### Body weight

Body weight was measured twice per week throughout the experiment. Rats in the cycled groups (3CAF:4CHOW and 5CAF:2CHOW) were weighed at the beginning and end of their weekly CAF cycles (Monday/Thursday and Monday/Saturday, respectively) in order to track weight gain on and off the diets. Accordingly, half the Chow and 7CAF groups were weighed with the 3CAF:4CHOW group (Monday/Thursday) and half were weighed with the 5CAF:2CHOW group (Monday/Saturday).

### Place/object tests

Place and object recognition memory was tested as described previously^9^ on CAF days 16–18 and CAF days 23–25. Testing took place during the light cycle (0700–1500 h) in a different room to where rats were housed. The square test arena was made of black acrylic (60 cm × 60 cm with 60 cm high walls) with a 4 × 4 grid marked on the floor. Behaviour was recorded using a video camera mounted directly above the arena. Rats were habituated to the empty arena in two 10-min sessions held on consecutive days. Testing began the following day for half the rats (*n* = 6/group) and on the next day for the other half (*n* = 6); this factor had no significant effect on recognition memory (*F*s < 1 for all main and interaction effects when including ‘start day’ as a factor in analyses). Place and object recognition tests were held on consecutive days and their order (place-object vs. object-place) was counterbalanced within each group.

Each test began with a 5-min familiarisation period in which rats explored two identical, commercially available objects (e.g., ceramic mugs, aluminium cans, tins of various shapes) located in two of the arena’s central quadrants. The rat was then removed and returned to the home cage for a 5-min retention phase, during which the objects and arena were cleaned with 50% ethanol. The rat was then returned to the arena for a 3-min test phase in which one of the objects was novel (object test) or one of the original objects was moved to a novel location (place test). Recognition memory was quantified as the extent to which rats explored the novel object relative to the familiar object (object test), or the object moved to a novel location relative to the object in a familiar location (place test). This was calculated as a recognition index, a proportion varying between 0 and 1 according to the following formula: Recognition index = novel exploration (s)/[novel exploration (s) + familiar exploration (s)]. Higher values on this index indicate greater exploration of the novel/novel-located object and better recognition memory, whereas values of 0.5 reflect equal exploration of both objects and an impairment in object or place recognition.

Place and object recognition memory was assessed on CAF day 16–18 (Test 1), and CAF day 23–25 (Test 2). Groups exposed to CAF diet (3CAF:4CHOW, 5CAF:2CHOW and 7CAF) were always tested while on CAF diet, in keeping with our previous studies^9,29^. Groups 3CAF:4CHOW and 5CAF:2CHOW were always tested at least 24-h into their weekly CAF cycle. The staggered introduction of CAF diet was designed so that total access converged on days 16, 17 and 18, when Test 1 was held. Test 2 occurred at different times for the CAF and cycled groups in order to match total CAF exposure at 23–25 days, given that total exposure began to diverge after Test 1. The interval between Tests 1 and 2 was therefore 6–9 days for groups 7CAF and 5CAF:2CHOW, and 13–15 for group 3CAF:4CHOW. Two subsets of group Chow received their Test 2 at equivalent times to the CAF groups to control for the difference in between-test intervals. A 2 × (2) mixed-ANOVA comparing place and object recognition between the two subsets of the chow group (tested 6–9 or 13–15 days after Test 1), the effects of test type, group subset, and their interaction were not significant (all *F* < 1). Therefore, it is unlikely that the interval between Test 1 and Test 2 altered performance in other groups.

### EchoMRI

Three days after Test 1, lean and fat mass were quantified by EchoMRI at the University’s Biological Resources Imaging Laboratory. Rats were placed in a plastic tube allowing minimal movement and entered into the scanner (~2 min/rat). At the time of testing, total cafeteria diet exposure was 18, 20, and 21 days for groups 3CAF:4CHOW, 5CAF:2CHOW and 7CAF groups, respectively, and groups 3CAF:4CHOW and 5CAF:2CHOW were on chow.

### Endpoint anthropometric, adiposity and hormone measures

Rats were euthanised during the 9^th^ week of the diet, when total days of CAF exposure was 25–26 days for group 3CAF:4CHOW, 31–32 days for group 5CAF:2CHOW, and 35–36 days for group 7CAF. Groups 3CAF:4CHOW and 5CAF:2CHOW were euthanised on CAF diet after a minimum of 24-h exposure to CAF. Rats were deeply anaesthetised with an intraperitoneal injection of a ketamine (100 mg/kg) and xylazine (15 mg/kg) cocktail. Body weight, naso-anal length, girth (at the xiphoid process) were measured, and liver as well as retroperitoneal and gonadal fat pads were weighed. The colour and shape of the liver was scored on an ordinal scale range from 0–3, as previously reported^29^. Blood glucose was recorded using a glucometer (AccuChek) after removing the tail tip with a sterile scalpel. Approximately 3 ml blood was collected by cardiac puncture prior to decapitation and aliquoted into two tubes containing 10ul EDTA, which were centrifuged (5-min, 4 °C, 6000 G) prior to collection of plasma for storage at −80 °C. Plasma insulin concentrations were determined using commercially available ELISA kits (Crystal Chem Inc.® Ultra Sensitive Rat Insulin ELISA kit) with samples run in duplicate and according to manufacturer’s instructions.

### Data analysis

All data analysis was performed using IBM SPSS Statistics (V24). Body weight, food intake, recognition memory (object and place) and endpoint measures were analysed using one-way or mixed group × time ANOVAs. Significant group main effects were followed by post-hoc pairwise comparisons applying Tukey’s HSD correction to control Type-1 error rate. The only exception was liver scores, which were taken on an ordinal scale ranging from 0-3^29^ and analysed using non-parametric Kruskal-Wallis and Kolmogorov-Smirnov tests. Statistical significance was set at *p* < 0.05. Levene’s tests indicated that no dependent variables violated the assumption of homogeneity of variance. Food intake analyses used cage as the unit of analysis (i.e., n = 4/group), though, for clarity, figures express energy intake on a per-rat basis (i.e., assuming equal consumption by dividing total energy intake for each cage by three). Place and object test videos were scored using ODLog© software (www.macropodsoftware.com) by a trained observer blind with respect to experimental group. Half the videos were cross-scored by a second trained observer unaware of experimental groups, with good reliability between the two scorers (intra-class correlation = 0.75). Only active investigation with the rat’s head oriented toward the object was scored as exploration; close proximity to or climbing on the object was not scored as exploration unless it was accompanied by active exploration of the object.

## Results

### Energy intake

**Figure 2:**
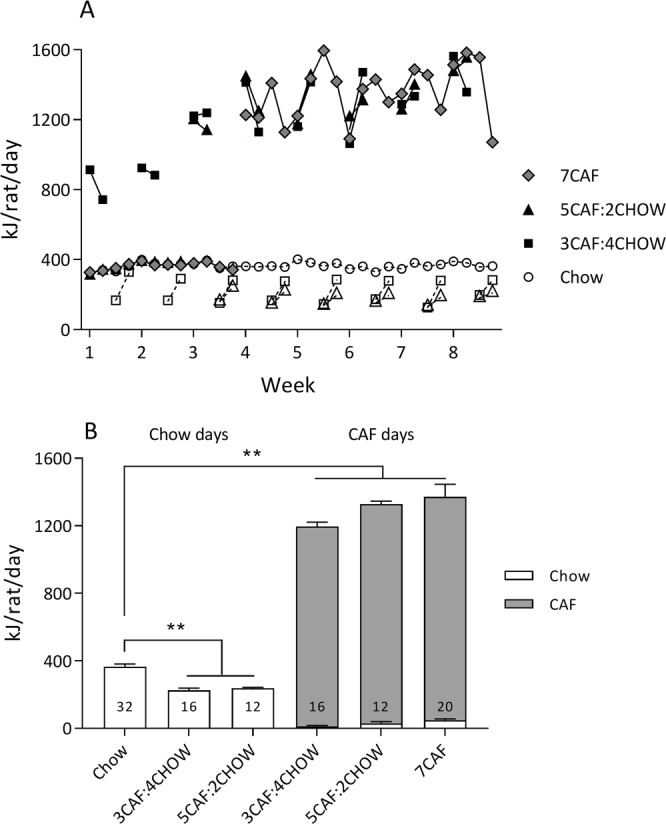
Energy intake. Four weekly measures of energy intake were taken across the experiment (Panel A). For cycled groups measures were taken on the first and last days of CAF (filled symbols) and chow access (unfilled symbols). CAF cycling began at week 1 for group 3CAF:4CHOW and at week 3 for group 5CAF:2CHOW; group 7CAF started at week 4. Panel B displays average energy intake on CAF days and chow days across the experiment. Numbers in columns indicate the number of measurements contributing to the average. **p < 0.01 versus Chow control group (Tukey HSD comparison following one-way ANOVA).

Energy intake across the experiment (4 measures/week) and collapsed group means are shown in Fig. 2A,B, respectively. Because the four groups differed in the number of measurements on chow versus CAF over the experiment, statistical analyses were applied to the overall means shown in Fig. 2B. A one-way ANOVA on average energy intake on CAF days found significant differences between groups (*F*(3, 12) = 114.68, *p* < 0.001). Post-hoc tests showed that energy intake was significantly higher in groups 3CAF:4CHOW, 5CAF:2CHOW and 7CAF than in the Chow control group (all *p* < 0.001) and that there were no significant differences among the three groups fed CAF (*F*’s < 1.0). A separate analysis, excluding the 7CAF group, found significant differences in energy intake on days when only chow was available for groups 3CAF:4CHOW, 5CAF:2CHOW and Chow control (one-way ANOVA *F*(2, 9) = 35.47, *p* < 0.001). Post-hoc tests showed that both cycled groups ate significantly less chow than the Chow control group (both *p* < 0.001) but did not differ significantly from each other (*p* = 0.801).

### Macronutrient intake

**Figure 3:**
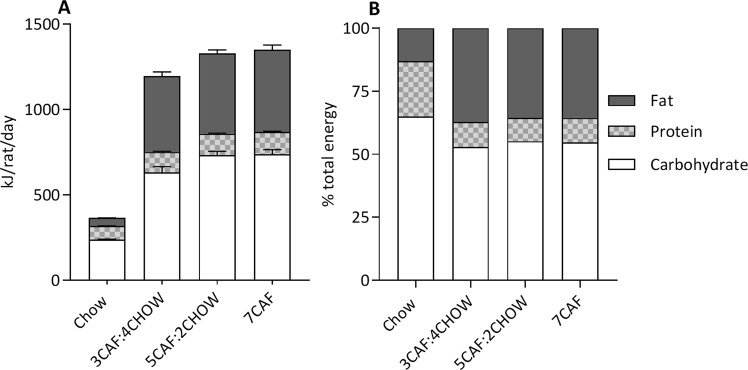
Macronutrient intake on days when CAF diet was available. Panel A: CAF diet access trebled energy intake and increased the absolute intake of all macronutrients. Panel B: When expressed as a proportion of total intake, CAF diet reduced the percentage of energy from protein and carbohydrate and increased the percentage from fat, relative to standard chow.

Figure 3A shows macronutrient intake averaged across the experiment on days when CAF diet was provided to groups 3CAF:4CHOW, 5CAF:2CHOW and 7CAF. There were significant differences between groups in the daily intake of carbohydrate (*F*(3, 12) = 53.53, *p* < 0.001), protein (*F*(3, 12) = 32.22, *p* < 0.001) and fat (*F*(3, 12) = 325.19, *p* < 0.001). Follow-up Tukey comparisons found intakes of each macronutrient were significantly elevated in all groups fed CAF relative to group chow (all *p* < 0.001), with no significant differences between the three CAF-fed groups (all *p* > 0.05). As shown in Fig. 3B, relative to group Chow, CAF diet exposure elevated the proportion of energy consumed as fat (37.2, 35.5 and 35.6% for groups 3CAF:4CHOW, 5CAF:2CHOW and 7CAF, respectively, versus 13% for group chow) and lowered the proportion of energy consumed as protein (9.9, 9.3 and 9.6 for groups 3CAF:4CHOW, 5CAF:2CHOW and 7CAF, respectively, versus 22% for group chow) and carbohydrate (53.0, 55.2 and 54.8% for groups 3CAF:4CHOW, 5CAF:2CHOW and 7CAF, respectively, versus 65% for group chow). Thus, whether CAF access was cycling or continuous did not alter the distribution of macronutrient intake. While total intake of each macronutrient was elevated by exposure to CAF diet, the proportion of total energy consumed as fat and protein was reduced. Thus, exposure to CAF diet increased total intake of each macronutrient but decreased the proportion of total energy consumed as protein and carbohydrate.

### Body weight on CAF day 18

**Figure 4:**
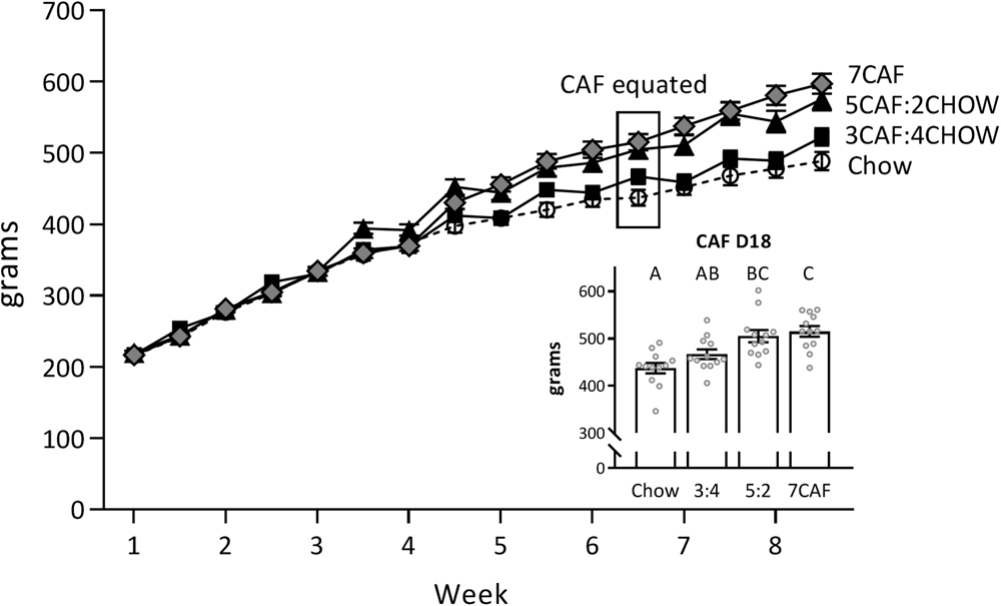
Body weight across the experiment. CAF diet increased body weight in a graded fashion according to the schedule of access. Inset shows group means after Test 1 when CAF access was matched at 18 days across groups. Groups not sharing a letter differ significantly at *p* < 0.05 (Tukey HSD comparison following one-way ANOVA).

Body weight across the experiment is displayed in Fig. 4. A group × time mixed-ANOVA found a significant linear increase in body weight over time (*F*(1, 44) = 2363.31, *p* < 0.001), a significant group × time linear interaction (*F*(3, 44) = 13.87, *p* < 0.001) and a significant group main effect (*F*(3, 44) = 3.93, *p* = 0.014). The key point of analysis was at the conclusion of Test 1, when total exposure to CAF diet converged at 18 days for all groups fed CAF (see Fig. 4, inset). A one-way ANOVA on body weights on CAF day 18 revealed significant differences between groups (*F*(3, 44) = 9.79, *p* < 0.001). Post-hoc Tukey tests suggested a graded effect of CAF schedule on body weight, such that the 7CAF group was significantly heavier than the Chow group (*p* < 0.001) and the 3CAF:4CHOW group (*p* = 0.023), whereas the 5CAF:2CHOW group was significantly heavier than the Chow group (*p* = 0.001) but did not differ significantly from the 3CAF:4CHOW group (*p* = 0.098). The 3CAF:4CHOW and Chow groups did not differ significantly (*p* = 0.280).

### Body composition at CAF day 18–21

**Figure 5:**
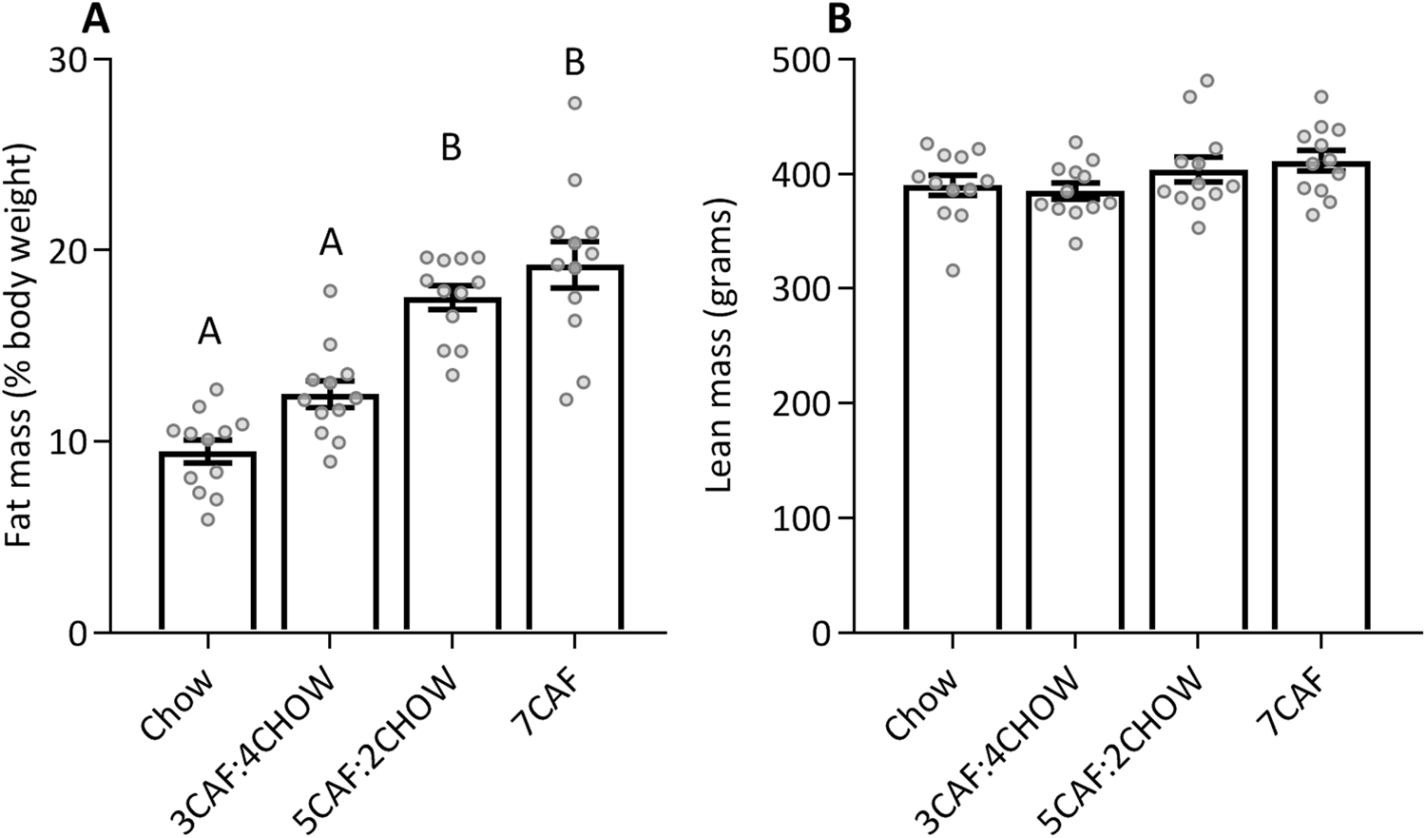
Body composition. Lean and fat mass were assessed by EchoMRI shortly after Test 1. Panel A depicts fat mass as percent total body weight; groups not sharing a letter differ significantly at *p* < 0.05 (Tukey HSD). Panel B shows absolute lean mass (grams).

Lean and fat mass was quantified by EchoMRI on the last day of week 6, when total CAF access was 18, 20 and 21 days for groups 3CAF:4CHOW, 5CAF:2CHOW and 7CAF, respectively. These data are shown in Fig. 5A (fat mass as percent body weight) and 5B (lean mass). Percent fat mass differed significantly between groups (*F*(3, 44) = 29.75, *p* < 0.001) and the pattern of differences were similar to those for body weight. Thus, fat mass was significantly higher in groups 5CAF:2CHOW and 7CAF than both groups 3CAF:4CHOW and Chow control (all *p* < 0.001) with no significant differences between either the two former groups (*p* = 0.462) or the two latter (*p* = 0.063). There were no significant between-group differences in absolute lean mass (one-way ANOVA: *F*(3, 44) = 1.82, *p* = 0.157), suggesting CAF access did not increase overall growth.

### Place task

**Figure 6:**
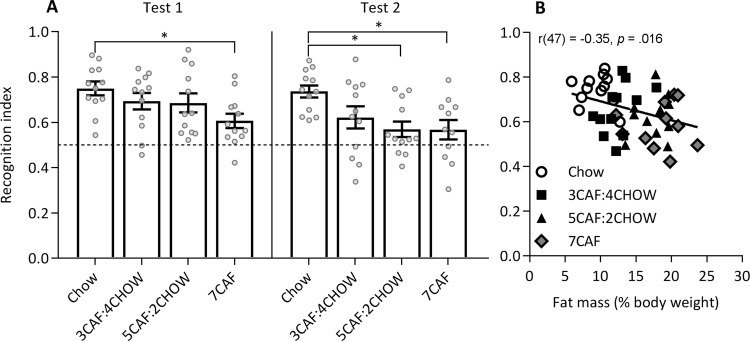
Place test results. Panel A: At Test 1 (left) place recognition memory was significantly poorer in the 7CAF group relative to Chow controls, whereas both 7CAF and 5CAF:2CHOW groups were impaired at Test 2 (right). * denotes *p* < 0.05 for pairwise comparisons using Tukey HSD correction. Figures show group mean (SEM) with individual data in grey circles. Panel B shows the negative correlation between percent fat mass (from EchoMRI) and average place recognition memory.

Results from the place task at Test 1 and Test 2 are displayed in the left and right panels of Fig. 6A, respectively. Test 2 data were excluded for one rat in the 7CAF group that failed to explore the objects during the test phase. At Test 1, one-way ANOVA of recognition index values indicated a trend toward significant differences between groups (*F*(3, 44) = 2.77, *p* = 0.053; Fig. 6A, left). Follow-up Tukey tests showed that recognition index values were significantly lower in the 7CAF group than the Chow control group (*p* = 0.032) but no other pairwise comparisons were statistically significant (all *p* > 0.32). One-sample *t*-tests indicated that all groups performed significantly better than chance (0.5) (group Chow *t*(11) = 8.15, *p* < 0.001; group 3CAF:4CHOW *t*(11) = 5.34, *p* < 0.001; group 5CAF:2CHOW *t*(11) = 4.45, *p* = 0.001; group 7CAF *t*(11) = 3.38, *p* = 0.006).

At Test 2 (Fig. 6A, right), the group main effect was significant (*F*(3, 43) = 4.18, *p* = 0.011) and pairwise comparisons found that relative to the Chow control group, place recognition memory was significantly lower in the 7CAF (*p* = 0.021) and 5CAF:2CHOW groups (*p* = 0.018), whereas the 3CAF:4CHOW group did not differ significantly from any group (smallest *p* = 0.168 vs. Chow control). One-sample *t*-tests showed that place recognition memory was significantly greater than chance (0.5) for the Chow and 3CAF:4CHOW groups (*t*(11) = 8.83, *p* < 0.001 and *t*(11) = 2.50, *p* = 0.03, respectively) but not 5CAF:2CHOW and 7CAF (*t*(11) = 2.05, *p* = 0.065 and *t*(10) = 1.58, *p* = 0.145, respectively).

A separate analysis evaluated changes in place recognition memory from Test 1 to Test 2 in a 4 × (2) mixed-ANOVA (group × [test]). This analysis found that place recognition decreased significantly from Test 1 to Test 2 (*F*(1, 43) = 5.33, *p* = 0.026) but that this decrease did differ between groups (group × test interaction, *F* < 1). Averaged across tests, the main effect of group was significant (*F*(3, 43) = 5.89, *p* = 0.002) and pairwise comparisons indicated that place recognition memory was worse in Groups 7CAF and 5CAF:2CHOW than Chow control (*p* = 0.001 and *p* = 0.022, respectively), with no other significant between-group differences (all *p* > 0.135).

### Object task

**Figure 7:**
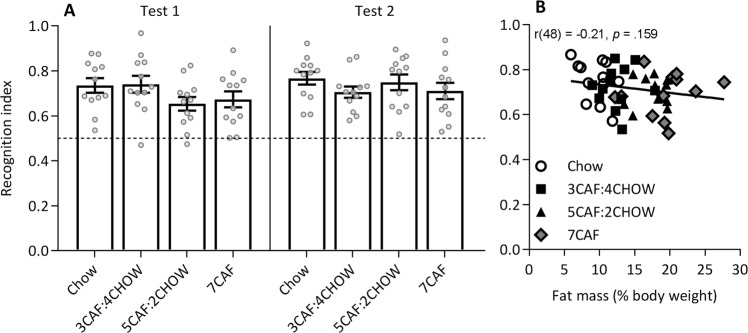
Object test results. No significant differences in object recognition were found at Test 1 (left) or Test 2 (right, panel A). Figure shows group mean (SEM) with individual rat data in grey circles. Panel B compares percent fat mass (from EchoMRI) and average object recognition memory.

Results from the object task at Tests 1 and 2 are shown in Fig. 7. There were no significant differences in object recognition memory at Test 1 (*F*(3, 44) = 1.66, *p* = 0.19) or Test 2 (*F* < 1). Combined analysis of the two tests indicated no significant change in performance over time (*F*(3, 44) = 2.32, *p* = 0.135), no group × time interaction (*F*(3, 44) = 1.60, *p* = 0.204) and no main effect of group (*F*(3, 44) = 1.10, *p* = 0.358). One-sample *t*-tests confirmed that recognition memory was significantly above chance (0.5) in all groups at both tests (all *t*(11) > 4.91, all *p* < 0.001).

**Figure 8:**
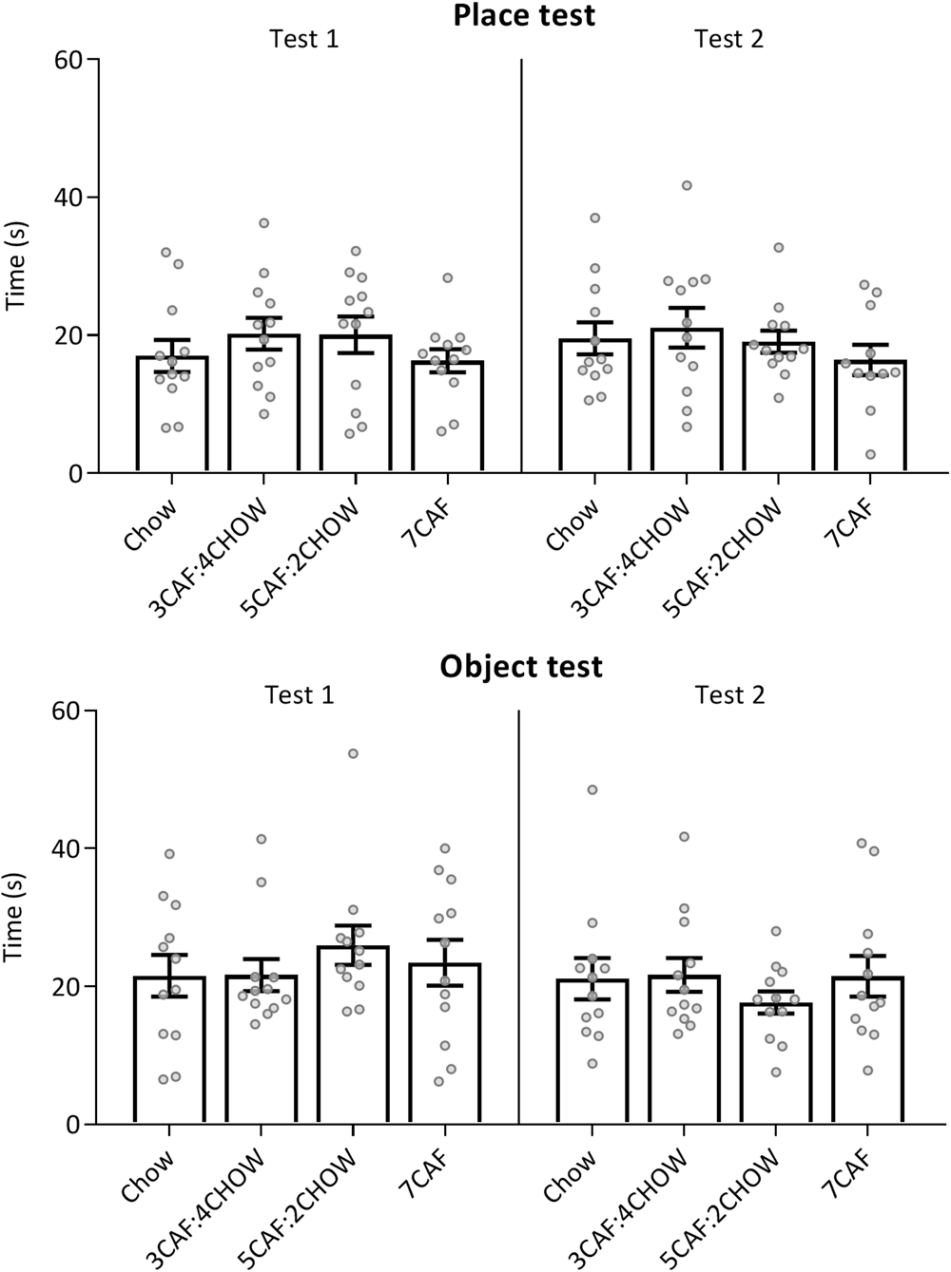
Total exploration time (s) in the place/object tests. Groups did not differ in total exploration time on either task at Test 1 or Test 2.

As shown in Fig. 8, there were no significant between-group differences in total exploration time on the place task (top panels; Test 1: *F* < 1; Test 2: *F*(3, 44) = 1.17, *p* = 0.33) or object tasks (bottom panels, both *F* < 1).

### Endpoint measures

As shown in Table 1, the dietary manipulations led to graded increases on body, organ and metabolic outcomes, with significant group differences found on every measure except blood glucose. Pairwise comparisons showed that continuous CAF exposure significantly increased body weight, length, girth, liver weight and score, white adipose tissue (WAT) mass and plasma insulin concentrations relative to Chow controls. The 5CAF:2CHOW group had significantly higher body weight and WAT mass, but not length, girth, or adjusted liver weight (g mass per kg body weight) than Chow controls. The 3CAF:4CHOW group had significantly greater liver scores, retroperitoneal and total WAT mass than Chow controls. Adiposity was more than doubled in 5CAF:2CHOW and 7CAF groups, with a 50% increase in 3CAF:4CHOW rats compared with chow controls. The profile of groups 3CAF:4CHOW and 5CAF:2CHOW diverged, with significant differences in body weight, nasoanal length and retroperitoneal, gonadal and total WAT. By contrast, groups 5CAF:2CHOW and 7CAF did not differ significantly on these measures, but girth and adjusted liver weight were significant greater in group 7CAF.

**Table 1:** Endpoint measures after cycling or continuous CAF diet.

Measure	Chow	3CAF:4CHOW	5CAF:2CHOW	7CAF	ANOVA *p*
Body weight (g)	503.0^A^ (13.3)	540.9^A^ (12.1)	602.8^B^ (17.6)	626.4^B^ (15.6)	<0.001
Nasoanal length (cm)	24.6^AB^ (0.2)	24.5^A^ (0.1)	25.3^BC^ (0.2)	25.4^C^ (0.2)	0.001
Girth (cm)	18.4^A^ (0.2)	18.7^A^ (0.3)	19.6^A^ (0.4)	21.2^B^ (0.5)	<0.001
Liver score (0–3)	0.08^A^ (0.1)	1.00^B^ (0.2)	1.83^BC^ (0.2)	2.50^C^ (0.2)	<0 0.001
Liver weight (g)	17.27^A^ (0.7)	18.40^AB^ (0.8)	21.18^BC^ (1.0)	24.30^C^ (0.9)	<0.001
Adj. liver weight (g/kg)	34.22^A^ (0.7)	33.95^A^ (1.2)	35.00^A^ (0.9)	38.72^B^ (0.9)	0.003
Retroperitoneal WAT (g)	6.63^A^ (0.7)	10.33^B^ (0.7)	16.86^C^ (0.6)	19.09^C^ (1.5)	<0.001
Gonadal WAT (g)	3.97^A^ (0.4)	7.05^A^ (0.6)	11.68^B^ (0.9)	13.26^B^ (1.4)	<0.001
Total WAT (g/kg)	20.87^A^ (1.8)	31.91^B^ (1.7)	47.51^C^ (2.2)	51.11^C^ (3.9)	<0.001
Blood glucose (mmol/L)	10.71 (0.5)	10.28 (0.3)	11.38 (0.5)	12.03 (0.7)	0.076
Plasma insulin (ng/ml)	0.41^A^ (0.1)	1.18^AB^ (0.3)	1.74^B^ (0.4)	2.18^B^ (0.4)	0.004

Values shown are group mean ± SEM. Significant group main effects from one-way ANOVAs (right column) were followed by pairwise comparisons applying the Tukey correction. Groups not sharing a letter differ significantly at p < 0.05. Liver scores analysed by nonparametric Kruskal-Wallis and Kolmogorov-Smirnov tests. Total WAT = sum of retroperitoneal and gonadal.

### Correlations

The average of place and object recognition memory scores across Tests 1 and 2 were each correlated with fat (% body weight) measured by EchoMRI, given that this was conducted in between Tests 1 and 2 when total CAF access was closely matched. Overall, higher body fat was associated with lower place recognition memory (*r*(47) = −0.35, *p* = 0.016), as shown in Fig. 6B, but not with object memory (*r*(48) = −0.21, *p* = 0.16), as shown in Fig. 7B. However, the correlation between place memory and fat mass was not significant within any single group. Fat collected at endpoint, standardised for body weight (g/kg) correlated negatively with place recognition (*r*(47) = −0.36, *p* = 0.014) but not with object recognition (*r*(48) = −0.11, *p* = 0.46), while total liver weight was negatively associated with both place and object recognition (*r*(47) = −0.37, *p* = 0.011 and *r*(48) = −0.365, *p* = 0.011, respectively). Interestingly, place and object recognition were weakly positively correlated (*r*(47) = 0.30, *p* = 0.044).

## Discussion

Adverse cognitive effects following access to diets high in saturated fats and refined carbohydrates have been demonstrated across a range of experimental procedures in rodents and humans. Such experiments have typically provided such diets *ad-libitum*, with less being known about the consequences of intermittent or ‘cycling’ exposure. Here, we report a comparison between continuous and cycling CAF exposure on cognitive and metabolic measures where cumulative access was matched between groups at test. Results indicated that the pattern of access to CAF diet, specifically, 3, 5 or 7 consecutive days per week, produced graded impairments in place recognition memory. Only continuous access impaired place memory at Test 1 (CAF day 16–18), but both 7CAF and 5CAF:2CHOW groups were impaired relative to Chow controls at Test 2, after a total of 23–25 days of CAF exposure.

The pattern of exposure to CAF diet exerted similarly graded effects on body weight and metabolic outcomes. Analysis of body composition conducted between Tests 1 and 2 revealed significantly greater fat mass in groups fed CAF for 5 or 7 days relative to those only fed chow or those given 3 days access to CAF diet, consistent with the pattern of place recognition memory deficits. These differences were maintained at the end point, when groups given 5 or 7 days CAF were significantly larger, fatter, and exhibited worse metabolic outcomes than Chow controls, with the 3CAF:4CHOW group intermediate. The overall negative correlation between fat mass and place recognition memory could suggest that the spread of low-grade inflammation from peripheral adipose tissue to the hippocampus, and other vulnerable brain regions, underlies the cognitive impairments observed here^30,31^, as found in past work from our laboratory^9^. Inflammation could also be increased directly by the diet itself rather than as a secondary consequence of excess adipose tissue^32^.

Among the most interesting aspect of the present results is the diverging profiles of the two cycling groups. By the end of the experiment Group 5CAF:2CHOW were significantly heavier, longer, and had greater fat mass than group 3CAF:4CHOW, and their metabolic ‘profile’ closely resembled the 7CAF group, differing on only 2 of the 11 endpoint measures collected (girth and adjusted liver weight). By contrast, group 3CAF:4CHOW differed from the 7CAF group on 9 of the 11 endpoint measures (all except blood glucose and plasma insulin), and only differed from group chow on adiposity measures (see Table 1). Our initial hypothesis was that place recognition memory would be comparable for the two cycled groups and that their recognition memory would be better than the poor memory in the continuous CAF group but worse than that in the chow group. In fact, only multiple cycles of five days CAF and two days chow led to detectable impairments in place recognition memory and worse metabolic outcomes, while three days of CAF and four days of chow did not. The two cycled groups did not differ either in their intake of CAF diet or in the extent of hypophagia when CAF diet was removed and replaced with chow, an effect shown in several past studies^14,28,33,34^. Unlike previous work^12,28,35^, however, cycling groups did not overeat CAF relative to the continuous CAF group, suggesting that the present design did not induce ‘binge’-like intake. This was unexpected in the group given the 3CAF:4CHOW schedule, which we previously demonstrated increased energy intake relative to continuous cafeteria diet exposure^28^. However, this may be because the present study used a more varied CAF diet (2–3 sweet and 2 savoury items each day, plus 10% sucrose solution) than that in the Martire *et al*. study, where rats always received 2 sweet and 2 savoury items but without the 10% sucrose solution. Thus, the additional variety and provision of sucrose solution appear to have fostered greater hyperphagia in the present study. Although consumption during the first two weeks of CAF appeared lower for the 3CAF:4CHOW group, suggesting lesser cumulative exposure to the diet, this was likely due to their lower body weight at this time. Indeed, adjusting energy intake for body weight (i.e. kJ/g body weight) corrected for this difference (see Supplementary materials). It will be important to test whether a 3CAF:4CHOW schedule has similar protective effects in animals outside of the growth phase when first exposed to CAF. However, unpublished work from our laboratory found that 3CAF:4CHOW cycling did not impair place recognition memory in weight-stable female rats (Leigh *et al*., in preparation), suggesting this is likely to be the case.

The fact that no differences were found in energy intake allow for the impairments in place recognition memory to be attributed to the pattern of access to CAF. In turn, this directs attention to several candidate mechanisms that could operate within the present durations of dietary exposures. One such mechanism is that any CAF diet-induced increase in hippocampal inflammation^9^ was blunted with 3-day cycling, sparing place recognition memory, relative to the well-documented inflammatory effects of continuous access and, presumably, of the longer 5-day cycling protocol. However, there is evidence that pro-inflammatory cytokines are upregulated in the hypothalamus very rapidly, within the first 24–72 hours of exposure to purified high-fat diets^36^. Moreover, such high-fat diets produce a milder metabolic and inflammatory phenotype than the cafeteria-style diets used here^37,38^. Taken together, these considerations suggest that increased inflammation could in fact be induced by the 3-day cycling protocol, raising the question as to why it failed to produce cognitive impairments. One possible answer is that the differential effects of repeated 3- and 5-day CAF cycles instead relate to the duration of time on chow (4 versus 2 days, respectively). The time-course of reported normalisation of diet-induced changes to neuronal structure and function varied: one study found that overnight withdrawal of a high-fat, high-sugar diet after 1-week of access reduced hypothalamic inflammation^39^, while another study found that high-fat diet-induced impairments in hippocampal plasticity were restored completely but over a 2-month period^40^. Given that the two cycling CAF groups differed with respect to both the CAF and chow portions of the cycle, results do not permit assessment of which portion was critical. Previous work has demonstrated that the impairment in place recognition produced by CAF diet feeding recovers within a week after switching to chow^41^.

Other work has shown that the permeability of the blood-brain barrier, which protects the CNS from circulating neurotoxins, is increased after 90^42^, but not 10 or 40 days of access to a high-fat diet^43^ suggesting that such changes in permeability were unlikely to have played a role in the present study. Markers of hippocampal inflammation, oxidative stress and blood-brain barrier integrity were not assessed here but will be critical to evaluate in future work on cycling access to unhealthy diets. The present study did not analyse such measures because Test 2 occurred on different days across groups, meaning that the interval between the final memory tests and endpoint varied between groups. It is important to note that while the introduction of the CAF diet was staggered and total days of access differed by the end of the experiment, all groups received equivalent handling, exposure to the colony room, and food intake measurements across the study. Finally, given evidence that the gut microbiome is profoundly affected both by dieting^44^ and by continuous^29^ or intermittent^45^ access to cafeteria diet, comparing the profile of the microbiome between the cycling schedules used here will be of interest given the cognitive effects observed.

A limitation of this study is that rats were group-housed, precluding analysis of individual energy intakes. While exploring CAF cycling with individually housed animals would permit the assessment of individual differences in the propensity to ‘binge’ and its relationship to the metabolic and cognitive effects of CAF diet, we avoided single housing due to potential stress-related impacts on behaviour. It will also be important to extend these findings to longer cycling periods to verify whether a ratio of 3 days CAF to 4 days chow progressively impairs place recognition memory. The apparent decrease in place memory from Test 1 to Test 2 for the 3CAF:4CHOW rats, although not statistically significant, nonetheless suggests that this schedule of access would ultimately impair place memory. Finally, given significant sex differences in dopaminergic and opioidergic gene expression following exposure to and subsequent removal of CAF diet^46^ and in the degree of cognitive impairment produced by high-sugar diets^47^, it will be important to reproduce the current design using female rats.

In summary, the present results show that the pattern of access to unhealthy diets is a critical determinant of their cognitive effects. Cycling access to CAF diet impaired place recognition memory only when access was provided for 5 or 7 consecutive days per week, controlling for total duration of exposure and with no differences in energy intake. This introduces the concept that, all else being equal, how consumption of different diets is distributed over time matters for long-term cognitive and physical health. Given the group differences observed over this relatively short-term study, it will be important for future research to continue to elucidate the minimum ‘concentration’ of access to unhealthy diets that poses risk for health over the long-term. The present findings add to existing evidence that even moderate restriction of consumption of high-fat, high-sugar foods is likely to make meaningful benefits to health.

## Supplementary information

Supplementary data

## Data Availability

The datasets generated and analysed during the current study are available from the corresponding author on reasonable request.
