# Supplementary material for: Pattern of access to cafeteria-style diet determines fat mass and degree of spatial memory impairments in rats
Source: Sci Rep. 2019 Sep 18;9:13516. doi: 10.1038/s41598-019-50113-3 (PMC6751189; doi:10.1038/s41598-019-50113-3)

Pattern of access to cafeteria-style diet determines fat mass and degree of spatial memory impairments in rats

Michael D. Kendig, R. Frederick Westbrook, & Margaret J. Morris

Supplementary material

Figure S1. Estimated total weekly energy intake. Energy intake was measured four times per week – two days on CAF and two days on chow for the cycled groups. These measures were used to estimate energy intake for each group over the entire week. For example, the 3CAF:4CHOW group's total energy intake was estimated as average daily CAF intake (based on two measures) multiplied by three, plus average daily chow intake (based on two measures) multiplied by four.

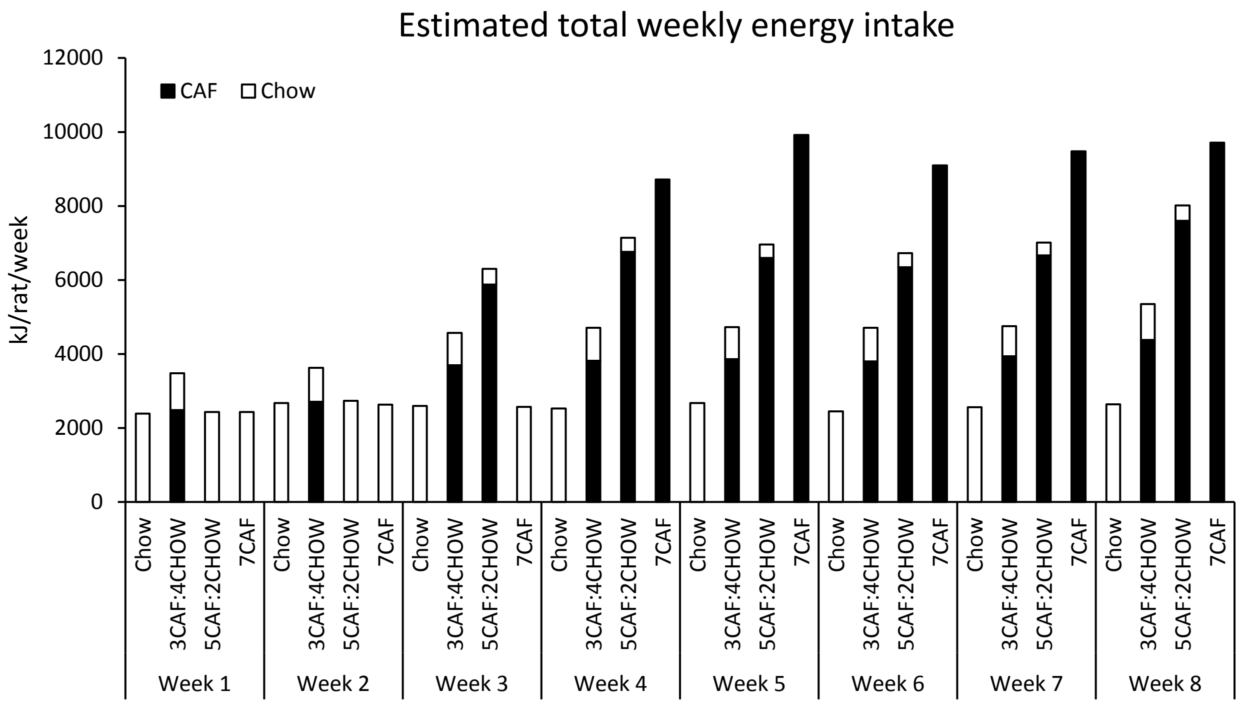

Figure S2. Energy intake (kJ) adjusted for body weight. Dividing consumption in kJ by body weight in grams (measured twice weekly) showed that consumption relative to body weight was comparable between groups.

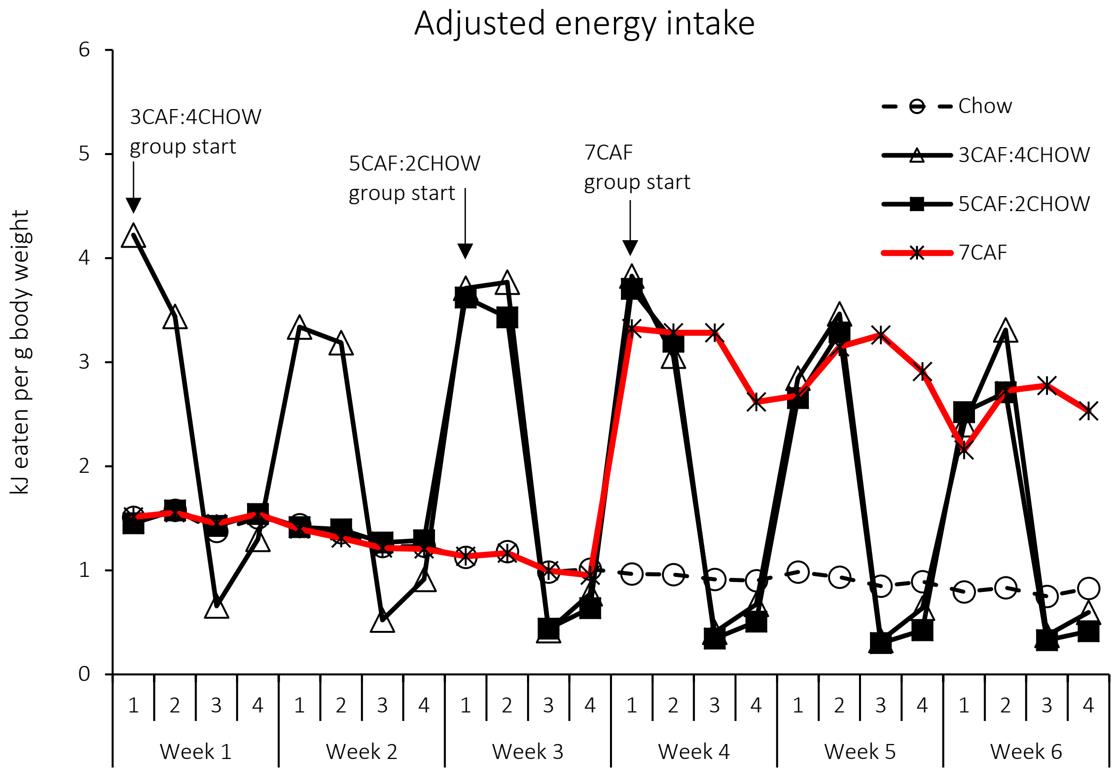

Supplement: Supplementary file 1 — Supplementary data [file 41598_2019_50113_MOESM1_ESM.pdf]
